# Puerarin combined with *Hericium erinaceus* insoluble dietary fiber alleviates obesity induced by high-fat diet through regulating the glycerophospholipid metabolism pathway influenced by gut microbiota

**DOI:** 10.1128/aem.02376-24

**Published:** 2025-02-20

**Authors:** Guoze Wang, Binbin Wang, Qin Zhou, Zhimei Cheng, Li Liu, Shuai Zhang, Shi Zhou, Peng Luo

**Affiliations:** 1 The Affiliated Hospital of Guizhou Medical University, Guizhou Provincial Engineering Research Center of Ecological Food Innovation, The Key Laboratory of Environmental Pollution Monitoring and Disease Control, Ministry of Education, School of Public Health, Guizhou Medical University 654239 , Guiyang, China; 2 The Key Laboratory of Precision Nutrition and Health of Ministry of Education, Harbin Medical University 34707 , Harbin, China; Universita degli Studi di Napoli Federico II, Portici, Italy

**Keywords:** *Hericium erinaceus*, insoluble dietary fiber, puerarin, untargeted metabolome, obesity, 16S rRNA

## Abstract

The purpose of this study was to evaluate the combined effects of puerarin (Pue) and insoluble dietary fiber from *Hericium erinaceus* (HEIDF) on obesity induced by a high-fat diet (HFD) in mice, focusing on their effects on lipid and glucose metabolism, gut microbiota (GM), and serum metabolites. Glucose tolerance, tissue pathology, and serum biochemical levels were conducted to assess the effects of puerarin combined with *Hericium Erinaceus* insoluble dietary fiber (LH) on glucose and lipid metabolism. 16S rRNA sequencing and untargeted metabonomics were employed to explore the underlying mechanisms. The results showed that the LH group significantly reduced body weight and hepatic and adipose lipid accumulation, and improved glucose tolerance and dyslipidemia compared to the Pue or HEIDF groups alone. Moreover, the LH group exhibited enhanced regulation of GM, including increased microbial diversity, higher abundance of beneficial bacteria such as g__Lactobacillus and g__Bacillus, and a decreased Firmicutes-to-Bacteroidota ratio. In addition, the LH group ameliorated HFD-induced serum metabolite changes and promoted the activation of tryptophan and glycerophospholipid metabolism pathways. The combination of Pue and HEIDF exhibits a synergistic anti-obesity effect by modulating specific GM (g__Lactobacillus and g__Bacillus) and serum metabolites.

**IMPORTANCE:**

The combination of HEIDF and Pue holds significant importance in the context of obesity. This synergistic effect not only aids in weight management but may also enhance metabolic health through various mechanisms, including increased satiety and promotion of fat oxidation. Therefore, incorporating these two components into the daily diet could offer effective strategies for the prevention and intervention of obesity and its related diseases.

## INTRODUCTION

*Pueraria lobata* is an edible plant with abundant nutritional and medicinal properties, and it is rich in various bioactive components. Polysaccharides and flavonoids are the major bioactive constituents of *Pueraria lobata*. Some studies have reported the anti-diabetic potential of its polysaccharide (1). Puerarin (Pue), one of the main isoﬂavonoid components, is known to possess antioxidant and anti-inﬂammatory properties and reduces insulin resistance among other effects (2). Using network pharmacology, molecular docking technology, and experimental verification, the research group found that puerarin inhibited cell proliferation and differentiation in a concentration-dependent manner, significantly promoted cell apoptosis, and influenced cell migration. Additionally, puerarin significantly inhibited the expression of AKT1, AKR1B1, MMP9, TNF, TP53, BCL2-2, and PPARG while significantly increasing the expression level of BAD protein in cell and animal models (3).

Dietary fiber (DF) consists of plant-derived carbohydrates that are indigestible and unabsorbable in the human small intestine. However, in the large intestine, DF undergoes anaerobic fermentation by certain gut microbiota (GM) (4, 5). DF is recognized as one of the seven essential nutrients. Additionally, studies have shown that increasing DF intake can reduce the incidence of diabetes, coronary heart disease, obesity, and various gastrointestinal disorders (6). DF can be categorized into soluble and insoluble dietary fiber (IDF). IDF, primarily composed of polysaccharides, is characterized by its loose and porous structure and rough surface. Research indicates that DF exhibits numerous physiological functions due to its excellent hydration, oil retention, and adsorption properties. These functions include regulating blood sugar levels, lowering blood lipids, preventing obesity, promoting intestinal health, facilitating defecation, and exhibiting antioxidant effects (7). IDF, a type of polysaccharide with a loose and porous structure and a rough surface, has been found to serve as non-covalent carriers for bioactive polyphenol compounds (8). The interactions between polyphenols and polysaccharides have notable effects on food processing. For instance, these interactions enhanced the antioxidant capacity of foods, modified the extractability of cell-wall polymers, reduced enzymatic susceptibility, and influenced fermentability (9). Changes in GM composition induced by polyphenol intake may further affect polysaccharide degradation pathways and energy metabolism. For example, the consumption of apples, which are rich in both polyphenols and dietary fibers, has been shown to alter the relative abundance of GM (10).

*Hericium erinaceus*, an important medicinal and edible fungus in China, is abundant in IDF, which has pharmacological effects such as anti-diabetes, anti-hypertension, and anti-hyperlipidemia (11). Nevertheless, this valuable fiber is often overlooked due to its less desirable taste. It may be discarded as a by-product during the extraction of active components like terpenoids or, alternatively, utilized as a substrate for fermentation. The soft texture of dietary fiber from *Hericium erinaceus* (HEIDF) potentially inhibits starch and fat digestion to a degree, thus potentially preventing obesity, although significant IDF waste still occurs (12).

Dietary changes play a significant role in the increasing prevalence of obesity and metabolic syndrome (13). Recently, high-fat diet (HFD) has contributed to a rapid rise in obesity and overweight, becoming a significant public health concern in China (14). An HFD not only induces obesity but also increases inflammatory responses, including GM disruption, endotoxemia, and adipose tissue inflammation, ultimately contributing to dyslipidemia (15). Numerous studies have shown that GM affects energy homeostasis, glucose and lipid metabolism, and related processes (16). Importantly, GM influences the production of various metabolites, some of which have been shown to prevent obesity and metabolic disorders (17).

We have previously conducted electron microscopy and infrared spectroscopy tests on the extracted HEIDF, confirming that the HEIDF obtained through this method possesses a porous honeycomb structure, is softer in texture, and has a stronger adsorption capacity for cholesterol and glucose compared to IDF extracted using other methods (18). Additionally, the research team discovered that adding insoluble dietary fiber from *Hericium erinaceus* can improve the release rate of puerarin and prolong its antioxidant activity by preparing HEIDF and Pue microcapsules. This approach enables targeted release in the intestine, thereby enhancing the bioavailability and bioefficacy of puerarin (19). Currently, it remains unclear whether the combined application of HEIDF and Pue in regulating dyslipidemia and glucose metabolism disorders is superior to individual interventions, both domestically and internationally. Therefore, we combined HEIDF and Pue to investigate their regulatory effects on lipid and glucose metabolism disorders induced by an HFD in mice. In this study, we measured the body weight, visceral fat weight, and glucose tolerance of HFD-induced obese mice and analyzed their fecal 16S rRNA and serum metabolites to uncover the potential mechanism by which Pue and HEIDF combination (LH) regulates lipid and glucose metabolism. This research not only ensures the full utilization of resources and minimizes waste but also expands the application fields and market potential of *Hericium erinaceus* and Pue, thereby creating additional benefits.

## RESULTS

### LH supplementation alleviates obesity-related traits provoked by HFD in mice

**Fig 1:**
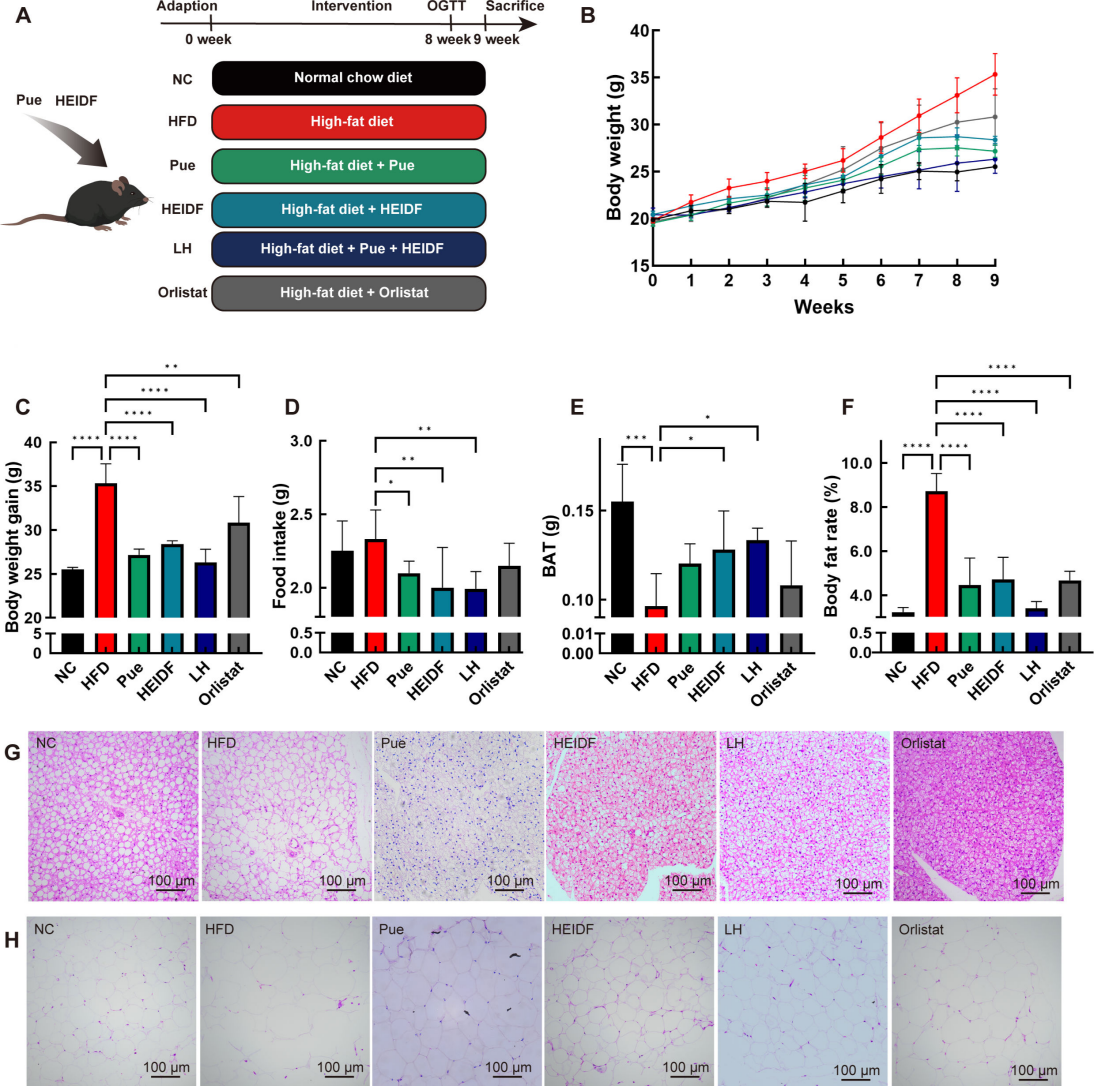
LH ameliorated obesity in HFD-fed mice. (**A**) Schematic diagram of the experiment design. (**B**) Body weight. (**C**) Body weight gain. (**D**) Food intake. (**E**) BAT. (**F**) Body fat rate. (G and H) Hematoxylin and eosin staining for BAT and eWAT (original magnification of 200×). Data are mean ± standard deviation. *P < 0.05, **P < 0.01, ***P < 0.001 vs the HFD group.

To study the protective mechanism of LH on metabolic disorder, we established an HFD-induced mouse obesity model and evaluated changes in parameters such as body weight, food consumption, and organ weight. After 9 weeks of HFD feeding, the body weight and final weight gain of mice in the HFD group were significantly higher than those in the normal control (NC) group. Following intervention with Pue or HEIDF, the body weight and final weight gain of the mice were notably reduced (Fig. 1B and C, *P* < 0.001), and food consumption was also significantly decreased (Fig. 1D, *P* < 0.05), with the LH group showing the most pronounced effect. Further analysis demonstrated that the LH group could reduce body weight gain by alleviating fat hypertrophy. The body fat percentage in the HFD group (*P* < 0.01) was higher than that in the NC group (Fig. 1E through H), while the percentage of brown adipose tissue (BAT) showed the opposite trend. Compared with the HFD group, supplementation with either Pue or HEIDF significantly reduced the changes in BAT percentage, body fat rate, and epididymal white adipose tissue (eWAT), as well as the size of lipid droplets and adipocytes in BAT. Among the groups, the LH group was more effective in reducing body weight and fat accumulation in mice. These results indicate that the LH group is more effective than a single intervention in reducing body weight in mice.

### LH ameliorates HFD-induced abnormal glucose tolerance

**Fig 2:**
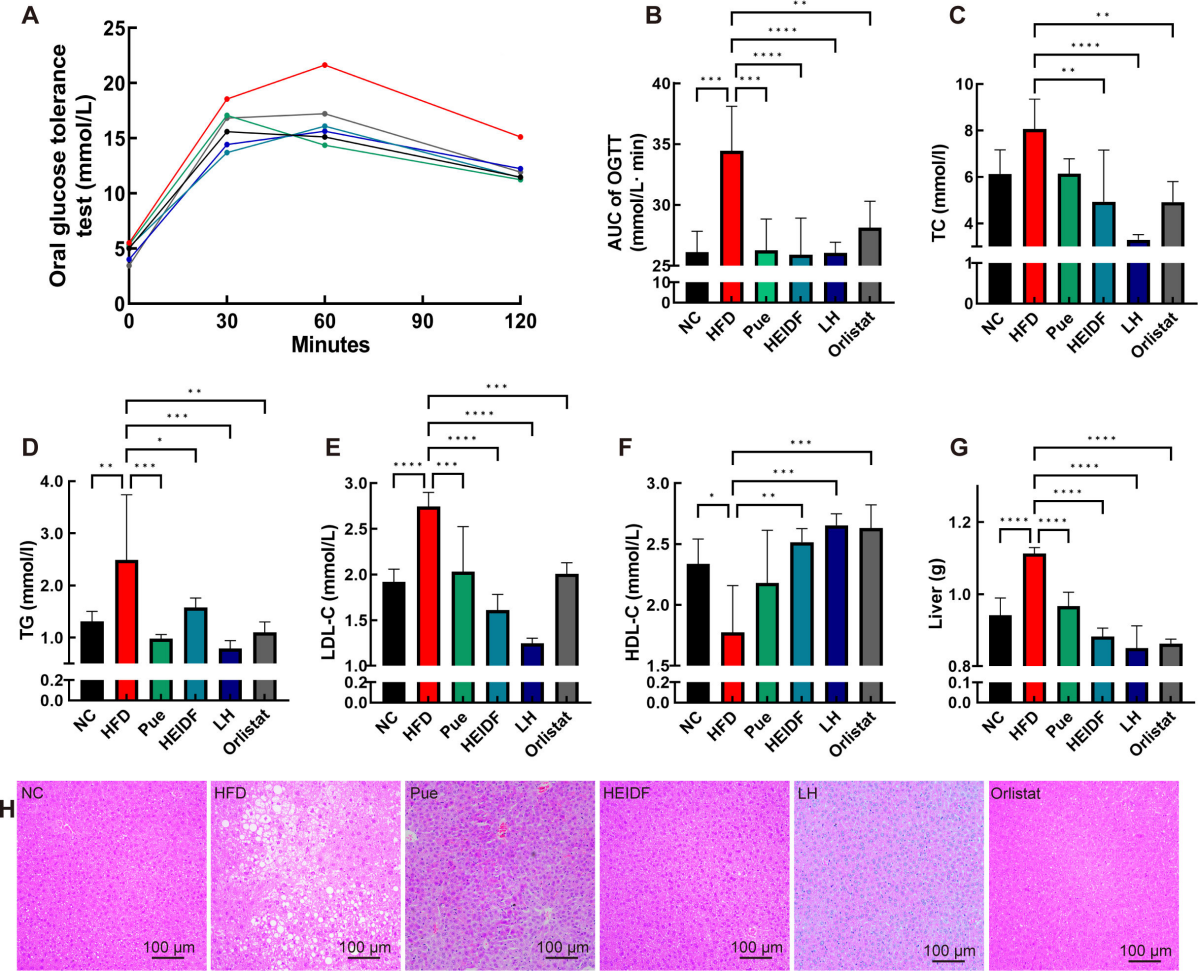
LH improved obesity-related metabolic diseases in mice fed with HFD. (**A**) OGTT. (**B**) AUC of OTGG. (C−F) Levels of total cholesterol (TC), triglyceride (TG), low-density lipoprotein cholesterol (LDL-C), and high-density lipoprotein cholesterol (HDL-C) in serum. (**G**) Liver weight. (**H**) Hematoxylin and eosin staining for liver (original magnification of 200×). Data are mean ± standard deviation. *P < 0.05, **P < 0.01, ***P < 0.001, ****P < 0.0001 vs the HFD group.

Past research has shown that mice fed an HFD can develop a range of metabolic disorders, including impaired glucose tolerance, lipid dysregulation, and liver damage (20, 21). The oral glucose tolerance test (OGTT) results revealed that glucose tolerance was significantly impaired in the HFD group compared to the NC group. However, this impairment was ameliorated in the Pue, HEIDF, and LH groups following interventions with Pue and HEIDF. Among these, the LH group demonstrated a greater improvement in glucose tolerance compared to the Pue and HEIDF groups (Fig. 2A). In this study, the LH group intervention also significantly reduced the area under the curve (AUC) of OGTT (Fig. 2B, *P* < 0.001). These results indicate that both the Pue and HEIDF groups improved HFD-induced glucose intolerance, and the LH group was more effective.

### LH ameliorates dyslipidemia and liver steatosis in HFD-fed mice

The serum levels of total cholesterol (TC), triglyceride (TG), and low-density lipoprotein cholesterol (LDL-C) in the HFD group were higher than those in the NC group, while high-density lipoprotein cholesterol (HDL-C) levels were lower (Fig. 2C through F, *P* < 0.05). However, there was no significant difference in TC levels between groups. The Pue, HEIDF, and LH groups showed a more significant alleviation of HFD-induced dyslipidemia compared to the Orlistat group. The LH group demonstrated the most pronounced alleviation effect, indicating that the combined intervention was superior to the single interventions in improving dyslipidemia (*P* < 0.001). The liver weight in the HFD group was markedly higher than that in the NC group (Fig. 2G, *P* < 0.001), whereas liver weights were effectively reduced by Pue and HEIDF intervention (*P* < 0.001). As shown in Fig. 2H, LH supplementation significantly decreased both liver weights and the incidence of hepatocyte ballooning and lipid droplets in the liver tissues of mice fed an HFD. These results indicate that the combined intervention using LH has a more substantial improvement effect on regulating dyslipidemia and liver injury induced by HFD.

### Effects of LH on GM composition in mice fed with HFD

**Fig 3:**
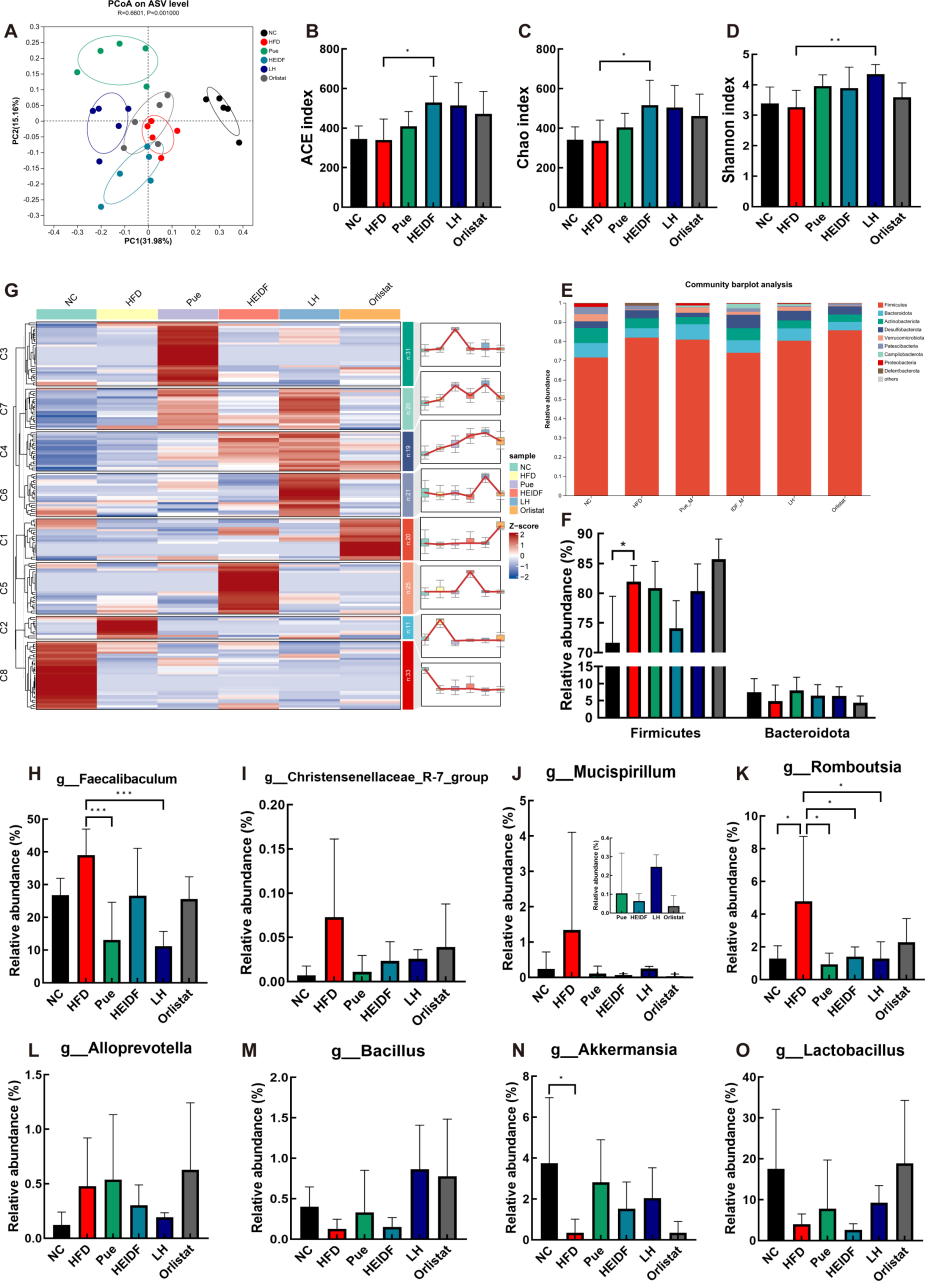
LH modulated the HFD-disrupted gut microbiota composition. (**A**) β-Diversity was analyzed and presented as a PCoA plot based on the amplicon sequence variant (ASV) level. (**B**) ACE indices. (**C**) Chao indices. (**D**) Shannon indices. (**E**) Relative abundance of community at phylum level. (**F**) Relative abundance of Firmicutes and Bacteroidota. (**G**) Clustering and *Z*-score trend of differentially expressed bacteria in each group (genus level). (H−O) Specific microbes regulated by LH. Data are mean ± standard deviation. *P < 0.05, **P < 0.01, ***P < 0.001 vs the HFD group.

Restoring gut dysbiosis is regarded as a crucial way through which dietary Pue or IDF can exert its beneficial effects (22, 23). Therefore, we conducted 16S rRNA sequencing to analyze the composition of the GM following treatment with LH. The principal coordinate analysis (PCoA) analysis showed a complete separation between the NC group and the HFD group (Fig. 3A). Furthermore, the HFD, Pue, and LH groups were completely separated, while the HFD and HEIDF groups partially overlapped, indicating that LH influenced the GM composition altered by HFD. For α-diversity analysis, abundance-based coverage estimator metric (ACE), Chao, and Shannon indices were used to evaluate the richness and diversity of GM. Compared with the NC group, ACE, Chao, and Shannon indices in the HFD group were substantially decreased (Fig. 3B through D). After LH intervention, the ACE and Chao indices in the LH group were increased. Among them, compared with the HFD group, the Shannon index of the LH group increased significantly. These results demonstrate that LH intervention increases the diversity of the GM.

### LH attenuates HFD-induced GM dysbacteriosis

The community composition of GM in each group at the phylum and genus levels was analyzed. At the phylum level, Firmicutes, Bacteroidota, Actinobacteriota, Desulfobacterota, and Verrucomicrobiota were the principal bacteria (Fig. 3E). In comparison to the NC group (7.49% and 71.65%, respectively), the Bacteroidota (4.91%) and Firmicutes (81.94%) were found to be higher in the HFD group, while the Pue, HEIDF, and LH groups increased Bacteroidota (8.04%, 6.48%, and 6.39%, respectively) and decreased Firmicutes (80.88%, 74.07%, and 80.32%, respectively), compared with the HFD group (Fig. 3F). At the genus level, cluster analysis of the relative abundance of GM revealed eight distinct clusters influenced by the different treatments (Fig. 3G). Specifically, compared with the NC group, the high-fat consumption downregulated the relative abundances of g__Akkermansia, g__Bacillus, and g__Lactobacillus but upregulated the relative abundances of g__Faecalibaculum, g__Mucispirillum, g__Christensenellaceae_R-7_group, g__Romboutsia, and g__Alloprevotella (Fig. 3H through O). The Pue, HEIDF, and LH groups alleviated HFD-induced gut dysbacteriosis, leading to a microbiota composition resembling that of the NC group. Additionally, the LH group exhibited a distinctive enhancement effect on the GM, particularly when compared to Pue and HEIDF groups. This effect was most pronounced in bacteria taxa associated with cluster 6, including g__Bacillus and g__Lactobacillus (Fig. 3M and O).

### The effect of LH on glycerophospholipid metabolism

**Fig 4:**
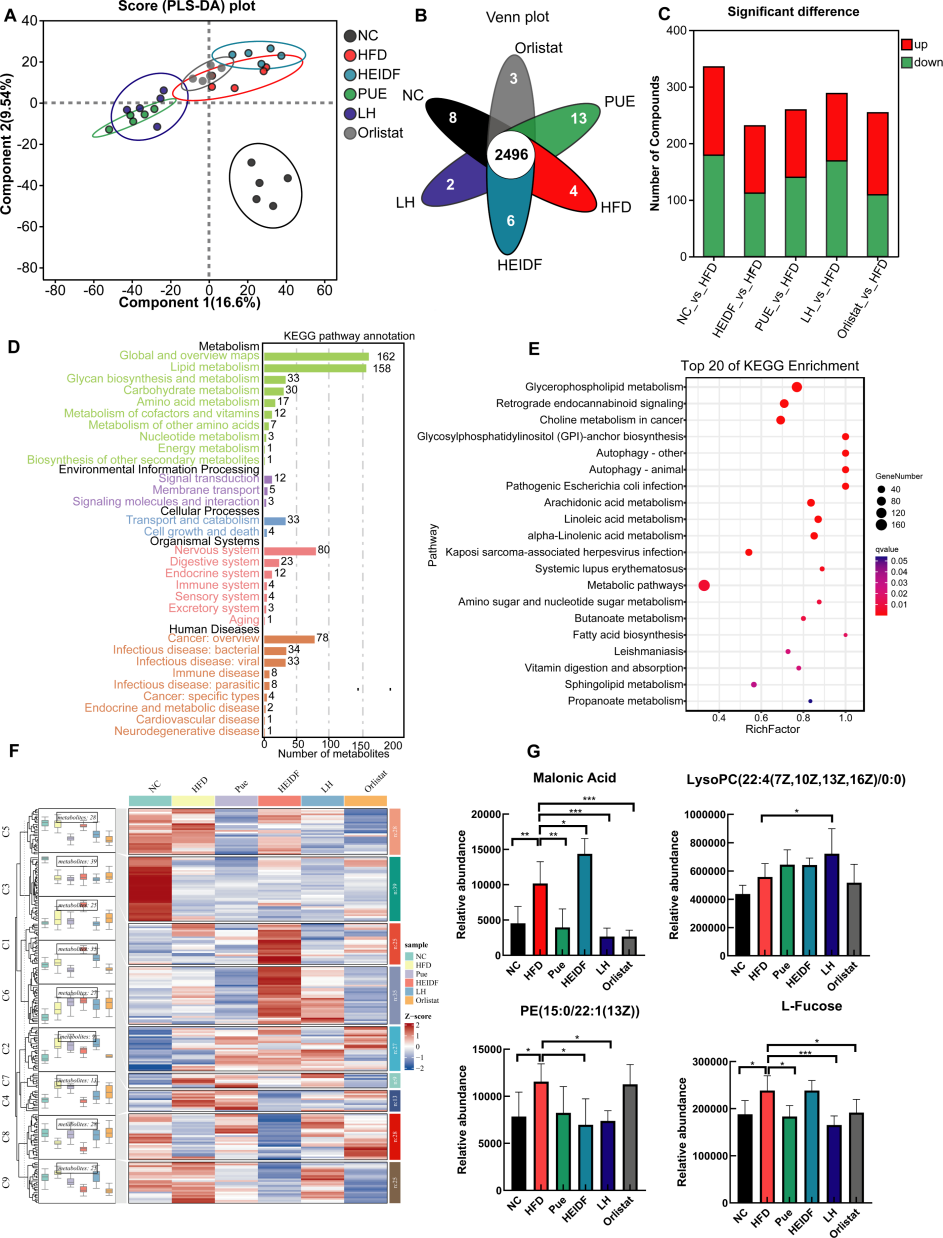
Metabolic effects of LH on the serum of HFD-fed mice. (**A**) PLS-DA. (**B**) Venn. (**C**) Stacked column chart of DEMs between groups. (**D**) KEGG pathway annotation. (**E**) Enrichment of KEGG. (**F**) Clustering and *Z*-score trend of metabolites in each group. (**G**) Comparison of DEMs between groups. *P < 0.05, **P < 0.01, ***P < 0.001 vs the HFD group.

A potential mechanism of the GM that is involved in host metabolism is closely linked to the metabolites produced through microbial fermentation within the gastrointestinal tract. In order to evaluate and quantify these metabolic alterations specifically induced by LH supplementation, UHPLC-QExactive metabolomics was employed to analyze the metabolic profile of serum samples from obese mice. This analysis aimed to further investigate the effects of LH on the serum metabolites of these mice. Totally, 2,688 metabolites were identified in the untargeted metabolomics analysis, representing 1,197 and 1,491 differential metabolites (DEMs) in the negative and positive ion modes, respectively. As shown in the partial least squares discriminant analysis (PLS-DA) plot (Fig. 4A), all group samples cluster significantly, with the NC group distinctly separating from the other groups. This indicates that there are considerable variations in the metabolites among the samples of each group, and the classification effect is notable. The Venn diagram reveals that there are 2,496 common metabolites among the groups (Fig. 4B). On the condition of variable importance in projection (VIP) values of >1.0 and *P* values of <0.05, the differential stacked bar chart showed that the LH group vs the HFD group comparison exhibited the highest number of DEMs relative to the other comparisons (Fig. 4C), with 119 upregulated DEMs and 169 downregulated DEMs. Based on the 229 DEMs that were related to obesity, we next conducted an analysis of the underlying metabolic pathways. Analysis of these pathways using the Kyoto Encyclopedia of Genes and Genomes (KEGG) database revealed five distinct categories: metabolism, environmental information processing, cellular processes, organismal systems, and human diseases. Interestingly, the global and overview maps and lipid metabolism were significantly enriched in the metabolism category (Fig. 4D). KEGG enrichment analysis (Fig. 4E) showed that the glycerophosphate metabolic pathway was the most significantly enriched. According to the trend clustering analysis (Fig. 4F), we identified DEMs that showed the same expression trend between the LH group and the NC group (C1 and C8). Among these, lysopc(22:4(7z,10z,13z,16z)/0:0), pe(15_0_22_1(13z)), l-fucose, and malonic acid were significantly involved in this pathway. According to Fig. 4G, the levels of these metabolites changed significantly in the LH group, and the intervention effect of the LH group was more pronounced than that of the Pue and HEIDF groups alone.

### Correlation analysis of “metabolites-microbial community indicators”

**Fig 5:**
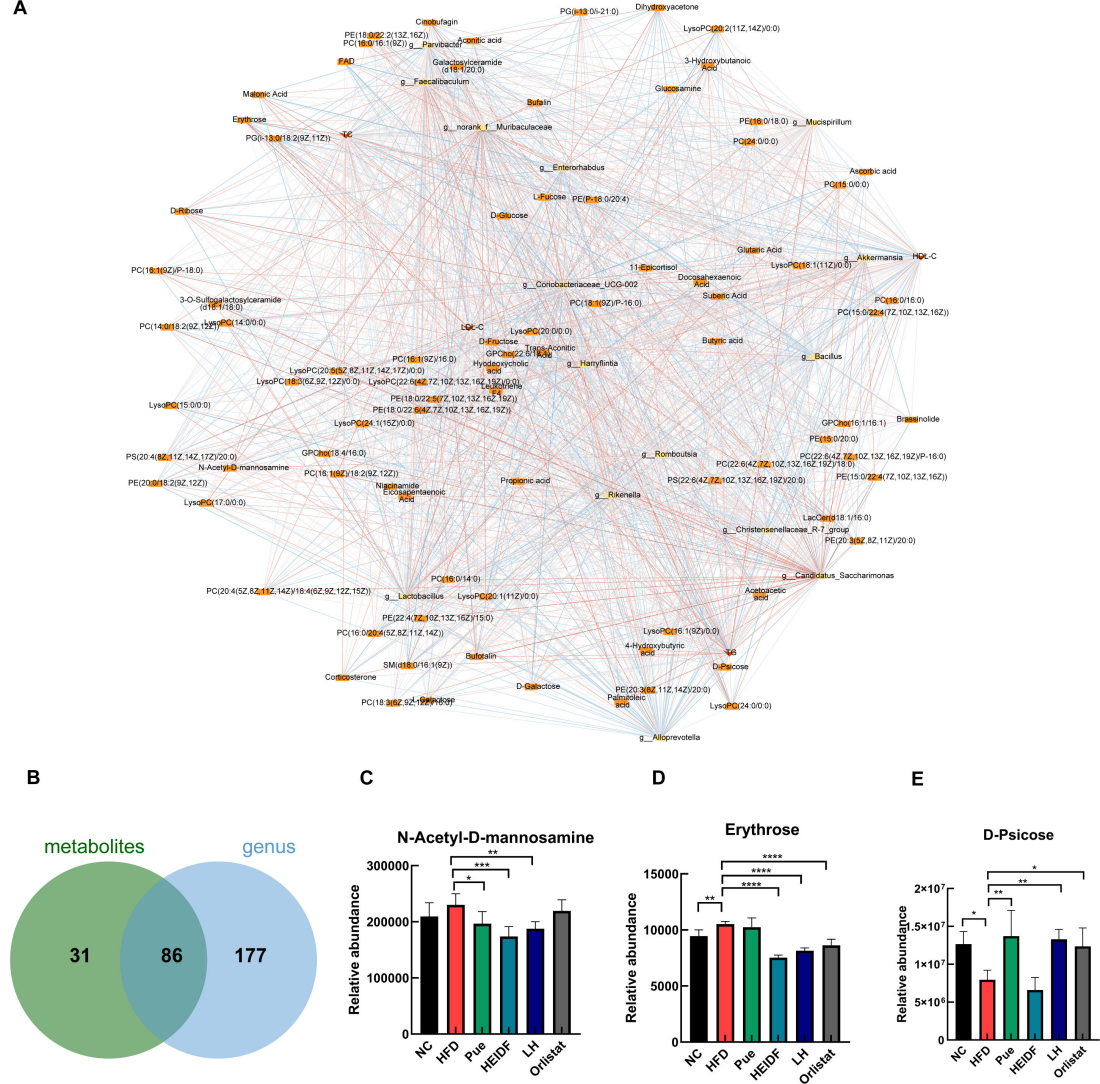
Combined effect of LH on non-targeted metabolism and 16S rRNA in HFD-fed mice. (**A**) “DEMs-GM indicators” network interactions. Ellipses represent GMs; squares represent DEMs; and triangles represent indicators. Red indicates positive correlation and blue indicates negative correlation. (**B**) Venn diagram of DEMs and genus co-enrichment pathway. (**C–E**) DEM expression levels between the different groups.

The correlations between GM and metabolites reflect the intricate interactions between microbial activity and host metabolism. For instance, GM can metabolize dietary components and produce bioactive compounds, such as short-chain fatty acids, which influence host lipid metabolism. Conversely, the host’s metabolic state can regulate the growth and function of specific microbial taxa, creating a dynamic feedback loop (24). Therefore, this study, which performed correlation network diagram analysis, showed (Fig. 5A) a positive correlation between bufotalin and g__Candidatus_Saccharimonas (|PCC| >0.8), while there was a negative correlation between D-psicose and g__Coriobacteriaceae_UCG-002–002 (|PCC| <0.6) and also a negative correlation between TG and g__Romboutsia, and the correlation between g__Mucispirillum and other factors was greater than 0.65, and LDL-C was positively correlated with erythrose and N-acetyl-D-mannosamine (|PCC| >0.75). In addition, there were 86 metabolic pathways in which metabolites and flora were co-enriched, such as fatty acid biosynthesis, glycerophospholipid metabolism, amino sugar and nucleotide sugar metabolism (Fig. 5B). Among them, lysopc(22:4(7z,10z,13z,16z)/0:0) and pe(15:0/22:1(13z)) were significantly involved in glycerophospholipid metabolism. According to Fig. 5C through E, the LH group increased the abundance of certain metabolites. Erythrose and n-acetyl-d-mannosamine in the HFD group were higher than those in the NC group, and the LH group was markedly lower than the HFD group after LH intervention. However, compared with the HFD group, d-psicose increased significantly in the LH group. Erythrose, n-acetyl-d-mannosamine, and d-psicose were involved in amino sugar and nucleotide sugar metabolism significantly. The results further demonstrate that LH can effectively change the richness of GM and the levels of beneficial DEMs, thereby activating metabolic pathways related to lipid metabolism and reducing lipid accumulation.

## DISCUSSION

Numerous studies show that the composition of GM is related to obesity and its metabolic-related diseases, and the joint analysis of GM and serum metabolites can more directly reveal the relationship between GM and host health (25, 26). However, there are few articles on the regulation of obesity lipid metabolism disorder by combining polyphenols with DF. Therefore, we established a mouse obesity model to reveal the regulatory effect of LH on lipid metabolism disorder. The results showed that LH could significantly reduce abnormal weight gain and lipid deposition, and improve blood lipid level and glucose tolerance level in obese mice induced by HFD, and the LH group showed more significant effects than those in the Pue and HEIDF groups alone.

The GM significantly influences the energy balance, nutrient uptake, and metabolic processes of the host organism. Related studies have demonstrated that the intake of an HFD reduces the richness and diversity of GM (27, 28). In the study, we sequenced the 16S rRNA from fecal samples of mice and compared the composition of GM among different groups. Previous research suggests that the ratio of Firmicutes to Bacteroidota (F:B) is a potential biomarker for obesity. Our findings indicate that the LH group exhibited significantly increased the diversity and richness of the GM. Compared to the HFD group, the LH group showed a reduced F:B and significant alterations in the relative abundance of genera such as g__Faecalibaculum, g__Lactobacillus, g__Bacillus, and g__Alloprevotella. Notably, the relative abundance of these genera in the LH group was higher than that in both the Pue and HEIDF groups. Research has highlighted that g__Lactobacillus plays various roles, including maintaining GM balance, improving digestive function, and enhancing immunity. In numerous disease models, g__Lactobacillus has been shown to ensure barrier integrity, mitigate inflammatory responses, and regulate hormone levels by activating the AhR pathway (29). Furthermore, g__Bacillus, particularly SC06, has been reported to significantly inhibit liver injury, as evidenced by improvements in liver structure, reductions in levels of ALT, AST, ALP and LDH, and the inhibition of mitochondrial dysfunction (30). Our study showed that the LH group decreased the relative abundance of g__Faecalibaculum and g__Alloprevotella while enhancing the relative abundance of g__Lactobacillus and g__Bacillus, and better promoted the production of tryptophan and other metabolic pathways.

GM can produce a variety of metabolites, including amino acids, short-chain fatty acids, vitamins (such as vitamins K and B), and secondary bile acids. These products have an important impact on the health of the host (31). For example, high glucose intake is related to the increase in intestinal inflammation and inflammatory bowel disease (32), so we have carried out non-targeted metabolomics detection on mice serum. We found that the LH group can better change the contents of metabolites related to lipid metabolism, such as lysopc(22:4(7z,10z,13z,16z)/0:0), pe(15_0_22_1(13z)), l-fucose, and d-psicose, and promote the activation of metabolic pathways such as the glycophorophospholipid metabolism pathway. Studies have shown that bufotalin can reduce the production of inflammatory factors in cells (33); d-psicose can impede fat accumulation by suppressing the manifestation of lipogenesis-related gene ACCα and hepatic fatty acid acquisition gene (SREBP-1c and FAS) while promoting the expression of genes involved in fatty acid oxidation, including PPARα, HSL, and AMPK2α (34).

In this study, we integrated fecal 16S rRNA sequencing with serum non-targeted metabolomics to investigate the relationship between GM and host metabolism. The analysis revealed significant differences in GM composition and metabolite levels between the LH and HFD groups. Lipid-related metabolites such as butyric acid, propionic acid, d-glucose, and FAD were inversely correlated with g__Coriobacteriaceae_UCG-002–002 and positively correlated with beneficial bacteria such as g__Candidatus_Saccharimonas. Moreover, both GM and DEMs were co-enriched in 86 metabolic pathways, including the 2-oxocarboxylic acid metabolism, AMPK signaling pathway, and alanine, aspartate, and glutamate metabolism. Notably, g__Lactobacillus and g__Bacillus were negatively correlated with TC and TG, which aligns with findings from Wu et al. (35). Studies have found that g__Lactobacillus may promote the production of metabolites related to the carbohydrate metabolism pathway, regulate glucose, or lower cholesterol level (36). Downes et al. found that g__Alloprevotella predominantly generates succinate and acetate, which have the ability to enhance the intestinal barrier and affect anti-inflammatory function (37). These findings collectively support the hypothesis that specific GM taxa contribute to metabolic regulation by producing metabolites that influence host pathways. This research demonstrated that the intervention of the LH group enhanced the contents of flavonoids, amino acids, sterols, and terpenoids while reducing the contents of fatty acids, steroids, and other compounds. These metabolic shifts were likely driven by GM-modulated pathways, highlighting the intricate interplay between microbial activity and host metabolism.

These results suggest that the LH group may alter lipid metabolism-related metabolites, such as acetoacetic acid, ascorbic acid, and D-psicose, by increasing the abundance of beneficial bacteria such as g__Lactobacillus and g__Bacillus. This activation could enhance lipid-related metabolic pathways, including glycerophospholipid metabolism and alanine, aspartate, and glutamate metabolism. Consequently, the LH group may help regulate lipid metabolism disorder induced by HFD, reduce abnormal weight gain, and improve the host’s glucose tolerance. However, while the correlation analysis highlights potential links, it does not establish causality. Moreover, the current study utilized non-targeted metabolomics and 16S rRNA sequencing, which, although powerful, have limitations in revealing direct causal mechanisms. To address these gaps, future studies will employ advanced techniques such as metagenomics, metabolic flux analysis, and targeted microbiome manipulation to further elucidate the precise interactions between gut microbiota and metabolites.

### Conclusion

In this study, the intervention with LH demonstrated significant effects on mitigating HFD-induced metabolic disorders, including reductions in body weight and improvements in blood glucose homeostasis, blood lipid profiles, and lipid metabolism in mice. The results revealed that LH supplementation modulated the GM-metabolite axis by enhancing the abundance of beneficial GM, such as g__Lactobacillus and g__Bacillus. These changes were associated with alterations in lipid metabolism-related serum metabolites, including acetoacetic acid, ascorbic acid, and d-psicose. Pathway analysis further highlighted the enrichment of lipid metabolism-related pathways, such as glycerophospholipid metabolism, alanine, aspartate, and glutamate metabolism, suggesting that these pathways may play a central role in LH’s regulatory effects on lipid metabolic disorders. Moreover, the integrated multi-omics analysis revealed a significant difference in microbiome abundance and metabolomics expression between the HFD and LH groups, suggesting that glycerophospholipid metabolism may be the key mechanism through which LH regulates lipid metabolism disorder. These findings not only advance our knowledge of the GM-metabolite axis but also offer potential strategies for preventing obesity and related metabolic diseases.

## MATERIALS AND METHODS

### Materials

The dried products were provided by Sansui County Forestry Ecological Development Co., Ltd. (Guizhou, China). Puerarin (#C14892164, 98% purity) was acquired from Shanghai Macklin Biochemical Technology Co., Ltd. (Shanghai, China). Purified high-fat feed (fat, carbohydrate, and protein energy supply ratio of 60%, 20%, and 20%) and control feed (fat, carbohydrate, and protein energy supply ratio of 35%, 26%, and 26%) were acquired from Jiangsu Xietong Pharmaceutical Bio-engineering Co., Ltd. (Jiangsu, China). Orlistat was acquired from Chongqing Huasen Pharmaceutical Co., Ltd. (Chongqing, China). The common assay kits of TG, TC, LDL-C, and HDL-C were purchased from Nanjing Jiancheng Bioengineering Institute (Nanjing, China).

### Extraction of IDF from HE

The extraction of IDF was adapted from established methods with slight refinements (38). The high-efficiency extraction of HEIDF was achieved through an ultrasonic-assisted enzymatic approach, outlined succinctly as follows: mix the powdered material with purified water (1:20); adjust the pH level to 6.0; and add 1.2% neutral protease. Apply ultrasonic treatment at 50°C, 200 W, for 2 h. Then, add 1.5% α-amylase, and repeat ultrasonic treatment at 65°C, 200 W, for 2 h. Deactivate enzymes by boiling for 10 min. Centrifuge at 5,000 rpm for 10 min; wash the precipitate with water; and freeze-dry the resulting IDF product. The extraction rate is 39.54% ± 1.56%.

### Preparation of HEIDF and Pue solutions

HEIDF or Pue was suspended in normal saline by continuous stirring to ensure uniform dispersion. For the combined administration of HEIDF and Pue, the Pue solution was mixed with the HEIDF suspension to form a homogeneous mixture before oral gavage. The suspension was freshly prepared daily before administration. Mice were orally gavaged with the HEIDF and Pue suspension at a dose of 0.2 mL/20 g body weight once daily for 9 weeks.

### Animal experiments

Male C57BL/6J mice (20 ± 2 g) were purchased from the Experimental Center of Guizhou Medical University. After a week of acclimation, mice were randomly divided into six groups (Fig. 1A): NC (*n* = 5); HFD (*n* = 5); mice maintaining HFD were administrated with 200 (HEIDF, *n* = 5), 400 (Pue, *n* = 5) and 200 (HEIDF) + 400 (Pue) (LH, *n* = 5) mg/kg B.W., respectively. The positive control (orlistat) received an HFD supplemented with 15.6 mg/kg B.W. of orlistat. Intervention lasted for 9 weeks. Dietary consumption and weight were recorded weekly.

After intervention, the mice fasted for 12 h before anesthesia. Blood samples were collected from eyeballs and transferred to sterile, enzyme-free centrifuge tubes. Post-centrifugation at 3,000 rpm for a duration of 15 min, the serum samples were obtained (Beijing Dalong, D1524R) and stored at −80°C. Subsequently, after cervical dislocation and euthanasia, fresh fecal, liver, and fat samples were promptly collected and then rapidly preserved in either −80°C or in 10% (vol/vol) neutral formalin solution for subsequent analysis.

### OGTT

An OGTT was performed according to the published report (39). During the penultimate week of the experimental period, all mice were subjected to a 12 h fasting period, followed by a 2 h OGTT. For this purpose, each mouse group was given a standardized dose of 2 g/kg of body weight through gavage. Subsequently, blood glucose levels were detected at 0, 30, 60, and 120 min post-gavage using tail-tip blood sampling. The AUC was determined to evaluate glucose tolerance and response.

### Examination of biochemical indicators

The blood samples collected after the mice were killed were left for 30 min, spun down at 4°C and 3,000 rpm/min in a frozen centrifuge (Beijing Dalong, D1524R) for 15 min to separate the upper serum, and immediately stored in liquid nitrogen until analysis. The serum levels of TG, TC, HDL-C, and LDL-C were determined utilizing commercial kits according to the manufacturer’s guidelines.

### Histopathological examinations

Fresh samples of liver, BATs, and eWATs were soaked in 10% neutral formalin solution for 24 h to stabilize and maintain their structural integrity. After fixation, these samples underwent embedding in paraffin and slicing. Paraffin-embedded sections of experimental samples were then stained with hematoxylin and eosin, visualized by optical microscope, and morphologically evaluated (×20).

### 16S rRNA sequencing and analysis

After total DNA extraction and DNA quality detection of mice stool samples, the extracted DNA was employed as a template. Primers 338F (5′-ACTCCTACGGGAGGCAGCA-3′) and 806R (5′-GGACTACHVGGGTWTCTAAT-3′) were used to amplify hypervariable region V3–V4 of the bacterial 16S rRNA gene. The PCR products were separated from 2% agarose gel and subsequently purified. The original reads were merged and trimmed, and then the chimera was removed. The raw reads underwent a process of merging and trimming, subsequently eliminating chimeras. After the equimolar amount of purified amplicon was collected, paired-end sequencing was performed on Illumina PE300 platform (Illumina, San Diego, USA). The classification and assignment of amplicon sequence variants were performed using the N Naive Bayes consensus taxonomy classifier, which was implemented in Qiime2 and utilized the SILVA 16S rRNA database (version 138). The above analysis was carried out by Majorbio Bio-Pharm Technology (Shanghai, China).

The α-diversity index, which includes the Shannon, ACE, and Chao indices, was used to analyze sequence data. We evaluated the β-diversity using the abund_jaccard and then visualized the results with PCoA. Furthermore, to delve into the functional characteristics of the GM, we utilized PICRUSt2 software (version 2.1.4) to forecast potential metabolic routes derived from the 16S sequencing data. Utilizing the KEGG (http://www.genome.jp/kegg), we conducted an in-depth analysis of the bacterial population structure and subsequently inferred the functional profiles of the microbiota.

### Non-targeted metabolomics analysis

Analytical measurements were conducted by Shanghai Majorbio Bio-Pharm Technology Co., Ltd. Serum samples were prepared as previously reported (40), and metabolite profiles were detected using UHPLC-QExactive (Ultimate 3000 LC, Q Exactive HF; Thermo-Fisher Scientific, Massachusetts, USA) with C18 column (Zorbax Eclipse C18 [1.8 µm × 2.1 mm × 100 mm]). The column temperature was kept at 40°C, with a flow rate of 0.4 mL/min. Mobile phase A was a 0.1% formic acid solution, whereas mobile phase B was pure acetonitrile in both negative and positive modes. The separation gradients were as follows: 0–0.1 min, linear increase of mobile phase B from 0% to 5%; 0.1–2.0 min, linear increase of mobile phase B from 5% to 25%; 2–9 min, linear increase of mobile phase B from 25% to 100%; 9–13 min, mobile phase B maintained at 100%; 13.0–13.1 min, linear decrease of mobile phase B from 100% to 0%; 13.1–16.0 min, mobile phase B maintained at 0%. The scanning mode included full scan (*m*/*z* 100–1500) and data-dependent mass spectrometry (MS) (dd-MS2, TopN = 10) with resolutions of 70,000 (MS1) and 17,500 (MS2). The collision mode was 20–40–60 V cycle collision energy.

Data were processed utilizing the Progenesis QI (Waters Corporation, Milford, USA). Metabolite annotation was conducted by matching MS and tandem mass spectrometry to Human Metabolome Database (http://www.hmdb.ca/), Metlin (https://metlin.scripps.edu/), and the self-defined Majorbio library.

### Statistical analysis

Data were presented as means ± standard deviation and were analyzed using GraphPad Prism (version 9.5; GraphPad Software, USA) and R (version 4.3.2). To assess statistical significance among more than two groups, one-way analysis of variance with Dunnett’s test was employed. This test facilitates the comparison of multiple treatment groups against a control group, enabling the identification of any significant differences among them. Furthermore, Spearman correlations between the GM and metabolites were calculated to explore the relationship between these variables. A *P* value of <0.05 was considered to be statistically significant, with *P < 0.05, **P < 0.01, and ***P < 0.001 vs the HFD group.

## Data Availability

All sequences used in this study are publicly available at the National Center for Biotechnology Information Sequence Read Archive (PRJNA1215323 Details | Manage Data | Submission Portal) under accession ID no. PRJNA1215323. All untargeted metabolomic data used in this publication have been deposited to the EMBL-EBI MetaboLights database with the identifier MTBLS12195 (serum metabolomics). The complete data set can be accessed at https://www.ebi.ac.uk/metabolights/MTBLS12195.

## References

[1] Luo D, Dong X, Huang J, Huang C, Fang G, Huang Y. 2021. Pueraria lobata root polysaccharide alleviates glucose and lipid metabolic dysfunction in diabetic db/db mice. Pharm Biol 59:382–390. doi:10.1080/13880209.2021.1898648 33794128 PMC8018507

[2] Zhou YX, Zhang H, Peng C. 2014. Puerarin: a review of pharmacological effects. Phytother Res 28:961–975. doi:10.1002/ptr.5083 24339367

[3] Mao J, Li M, Wang X, Wang B, Luo P, Wang G, Guo X. 2024. Exploring the mechanism of Pueraria lobata (Willd.) Ohwi in the regulation of obesity. J Ethnopharmacol 335:118703. doi:10.1016/j.jep.2024.118703 39154668

[4] Dong J-L, Wang L, Lü J, Zhu Y-Y, Shen R-L. 2019. Structural, antioxidant and adsorption properties of dietary fiber from foxtail millet (Setaria italica) bran. J Sci Food Agric 99:3886–3894. doi:10.1002/jsfa.9611 30684279

[5] de Menezes EW, Giuntini EB, Dan MCT, Sardá FAH, Lajolo FM. 2013. Codex dietary fibre definition - Justification for inclusion of carbohydrates from 3 to 9 degrees of polymerisation. Food Chem 140:581–585. doi:10.1016/j.foodchem.2013.02.075 23601410

[6] Wang G, Wang Y, Wang B, Su M, Zhou S, Luo P, Chen L. 2023. Prevention and control effects of edible fungi and their active ingredients on obesity: an updated review of research and mechanism. J Funct Foods 107:105621. doi:10.1016/j.jff.2023.105621

[7] Korczak R, Kamil A, Fleige L, Donovan SM, Slavin JL. 2017. Dietary fiber and digestive health in children. Nutr Rev 75:241–259. doi:10.1093/nutrit/nuw068 28586481

[8] Dobson CC, Mottawea W, Rodrigue A, Buzati Pereira BL, Hammami R, Power KA, Bordenave N. 2019. Impact of molecular interactions with phenolic compounds on food polysaccharides functionality. Adv Food Nutr Res 90:135–181. doi:10.1016/bs.afnr.2019.02.010 31445595

[9] Zhu F. 2018. Interactions between cell wall polysaccharides and polyphenols. Crit Rev Food Sci Nutr 58:1808–1831. doi:10.1080/10408398.2017.1287659 28362107

[10] Wani TA, Shah AG, Wani SM, Wani IA, Masoodi FA, Nissar N, Shagoo MA. 2016. Suitability of different food grade materials for the encapsulation of some functional foods well reported for their advantages and susceptibility. Crit Rev Food Sci Nutr 56:2431–2454. doi:10.1080/10408398.2013.845814 25603446

[11] Khan MA, Tania M, Liu R, Rahman MM. 2013. Hericium erinaceus: an edible mushroom with medicinal values. J Complement Integr Med 10:253–258. doi:10.1515/jcim-2013-0001 23735479

[12] Chong PS, Fung M-L, Wong KH, Lim LW. 2020. Therapeutic potential of Hericium erinaceus for depressive disorder. IJMS 21:163. doi:10.3390/ijms21010163 PMC6982118 31881712

[13] Kawano Y, Edwards M, Huang Y, Bilate AM, Araujo LP, Tanoue T, Atarashi K, Ladinsky MS, Reiner SL, Wang HH, Mucida D, Honda K, Ivanov II. 2022. Microbiota imbalance induced by dietary sugar disrupts immune-mediated protection from metabolic syndrome. Cell 185:3501–3519. doi:10.1016/j.cell.2022.08.005 36041436 PMC9556172

[14] Geng J, Ni Q, Sun W, Li L, Feng X. 2022. The links between gut microbiota and obesity and obesity related diseases. Biomed Pharmacother 147:112678. doi:10.1016/j.biopha.2022.112678 35134709

[15] Kim K-A, Gu W, Lee I-A, Joh E-H, Kim D-H. 2012. High fat diet-induced gut microbiota exacerbates inflammation and obesity in mice via the TLR4 signaling pathway. PLoS One 7:e47713. doi:10.1371/journal.pone.0047713 23091640 PMC3473013

[16] Canfora EE, Meex RCR, Venema K, Blaak EE. 2019. Gut microbial metabolites in obesity, NAFLD and T2DM. Nat Rev Endocrinol 15:261–273. doi:10.1038/s41574-019-0156-z 30670819

[17] Mo X, Sun Y, Liang X, Li L, Hu S, Xu Z, Liu S, Zhang Y, Li X, Liu L. 2022. Insoluble yeast β-glucan attenuates high-fat diet-induced obesity by regulating gut microbiota and its metabolites. Carbohydr Polym 281:119046. doi:10.1016/j.carbpol.2021.119046 35074119

[18] Wang BB, Li ML, Wang S, Wang Y, Guo XL, Luo P, Wang GZ. 2024. Effects of extraction methods on structure, physicochemical and functional properties of insoluble dietary fiber from Hericium erinaceus. Food Res Dev 45:14–22. doi:10.12161/j.issn.1005-6521.2024.17.003

[19] Li ML, Wang S, Wang Y, Pu HX, Tang RY, Guo XL, Wang GZ. 2024. Puerarin-Hericium insoluble dietary fiber compound preparation of microcapsule and study of the physical and chemical properties. Sci Technol Food Ind 45:71–80. doi:10.13386/j.issn1002-0306.2023080261

[20] Zeng SL, Li SZ, Xiao PT, Cai YY, Chu C, Chen BZ, Li P, Li J, Liu EH. 2020. Citrus polymethoxyflavones attenuate metabolic syndrome by regulating gut microbiome and amino acid metabolism. Sci Adv 6:eaax6208. doi:10.1126/sciadv.aax6208 31922003 PMC6941918

[21] Su J, Liu X, Li H, Cheng X, Shi S, Li N, Wu J, Xu Y, Liu R, Tian X, Wang H, Wang S. 2020. Hypoglycaemic effect and mechanism of an RG-II type polysaccharide purified from Aconitum coreanum in diet-induced obese mice. Int J Biol Macromol 149:359–370. doi:10.1016/j.ijbiomac.2020.01.209 31981662

[22] Yang CW, Liu HM, Chang ZY, Liu GH, Chang HH, Huang PY, Lee TY. 2024. Puerarin modulates hepatic farnesoid X receptor and gut microbiota in high-fat diet-induced obese mice. Int J Mol Sci 25:5274. doi:10.3390/ijms25105274 38791314 PMC11121391

[23] Liu Z, Dai J, Liu R, Shen Z, Huang A, Huang Y, Wang L, Chen P, Zhou Z, Xiao H, Chen X, Yang X. 2024. Complex insoluble dietary fiber alleviates obesity and liver steatosis, and modulates the gut microbiota in C57BL/6J mice fed a high-fat diet. J Sci Food Agric 104:5462–5473. doi:10.1002/jsfa.13380 38348948

[24] Ahmed H, Leyrolle Q, Koistinen V, Kärkkäinen O, Layé S, Delzenne N, Hanhineva K. 2022. Microbiota-derived metabolites as drivers of gut-brain communication. Gut Microbes 14:2102878. doi:10.1080/19490976.2022.2102878 35903003 PMC9341364

[25] Yi X, Cai R, Shaoyong W, Wang G, Yan W, He Z, Li R, Chao M, Zhao T, Deng L, Yang G, Pang W. 2023. Melatonin promotes gut anti-oxidative status in perinatal rat by remodeling the gut microbiome. Redox Biol 65:102829. doi:10.1016/j.redox.2023.102829 37527604 PMC10407234

[26] Le Roy T, Lécuyer E, Chassaing B, Rhimi M, Lhomme M, Boudebbouze S, Ichou F, Haro Barceló J, Huby T, Guerin M, Giral P, Maguin E, Kapel N, Gérard P, Clément K, Lesnik P. 2019. The intestinal microbiota regulates host cholesterol homeostasis. BMC Biol 17:94. doi:10.1186/s12915-019-0715-8 31775890 PMC6882370

[27] Ye L, Zhang Q, Xin F, Cao B, Qian L, Dong Y. 2021. Neonatal milk fat globule membrane supplementation during breastfeeding ameliorates the deleterious effects of maternal high-fat diet on metabolism and modulates gut microbiota in adult mice offspring in a sex-specific way. Front Cell Infect Microbiol 11:621957. doi:10.3389/fcimb.2021.621957 33816333 PMC8017235

[28] Fan Y, Pedersen O. 2021. Gut microbiota in human metabolic health and disease. Nat Rev Microbiol 19:55–71. doi:10.1038/s41579-020-0433-9 32887946

[29] Huang Z, Xie L, Huang L. 2023. Regulation of host immune responses by Lactobacillus through aryl hydrocarbon receptors. Med Microecol 16:100081. doi:10.1016/j.medmic.2023.100081

[30] Wu Y, Wang B, Tang L, Zhou Y, Wang Q, Gong L, Ni J, Li W. 2022. Probiotic Bacillus alleviates oxidative stress-induced liver injury by modulating gut-liver axis in a rat model. Antioxidants (Basel) 11:291. doi:10.3390/antiox11020291 35204173 PMC8868294

[31] Cryan JF, O’Riordan KJ, Sandhu K, Peterson V, Dinan TG. 2020. The gut microbiome in neurological disorders. Lancet Neurol 19:179–194. doi:10.1016/S1474-4422(19)30356-4 31753762

[32] Kawabata K, Kanmura S, Morinaga Y, Tanaka A, Makino T, Fujita T, Arima S, Sasaki F, Nasu Y, Tanoue S, Hashimoto S, Ido A. 2019. A high‑fructose diet induces epithelial barrier dysfunction and exacerbates the severity of dextran sulfate sodium‑induced colitis. Int J Mol Med 43:1487–1496. doi:10.3892/ijmm.2018.4040 30628636

[33] Park SJ, Jung HJ. 2023. Bufotalin suppresses proliferation and metastasis of triple-negative breast cancer cells by promoting apoptosis and inhibiting the STAT3/EMT axis. Molecules 28:6783. doi:10.3390/molecules28196783 37836626 PMC10574664

[34] Chen J, Huang W, Zhang T, Lu M, Jiang B. 2019. Anti-obesity potential of rare sugar d-psicose by regulating lipid metabolism in rats. Food Funct 10:2417–2425. doi:10.1039/c8fo01089g 30964474

[35] Wu Y, Zhang Q, Ren Y, Ruan Z. 2017. Effect of probiotic Lactobacillus on lipid profile: a systematic review and meta-analysis of randomized, controlled trials. PLoS One 12:e0178868. doi:10.1371/journal.pone.0178868 28594860 PMC5464580

[36] Diez-Gutiérrez L, San Vicente L, Sáenz J, Barron LJR, Chávarri M. 2022. Characterisation of the probiotic potential of Lactiplantibacillus plantarum K16 and its ability to produce the postbiotic metabolite γ-aminobutyric acid. J Funct Foods 97:105230. doi:10.1016/j.jff.2022.105230

[37] Downes J, Dewhirst FE, Tanner ACR, Wade WG. 2013. Description of Alloprevotella rava gen. nov., sp. nov., isolated from the human oral cavity, and reclassification of Prevotella tannerae Moore et al. 1994 as Alloprevotella tannerae gen. nov., comb. nov. Int J Syst Evol Microbiol 63:1214–1218. doi:10.1099/ijs.0.041376-0 22753527 PMC3709537

[38] Jia F, Liu X, Gong Z, Cui W, Wang Y, Wang W. 2020. Extraction, modification, and property characterization of dietary fiber from Agrocybe cylindracea. Food Sci Nutr 8:6131–6143. doi:10.1002/fsn3.1905 33282264 PMC7684601

[39] Jiao W, Sang Y, Wang X, Wang S. 2023. Metabonomics and the gut microbiome analysis of the effect of 6-shogaol on improving obesity. Food Chem 404:134734. doi:10.1016/j.foodchem.2022.134734 36327507

[40] Ren Y, Yu G, Shi C, Liu L, Guo Q, Han C, Zhang D, Zhang L, Liu B, Gao H, et al.. 2022. Majorbio Cloud: a one-stop, comprehensive bioinformatic platform for multiomics analyses. Imeta 1:e12. doi:10.1002/imt2.12 38868573 PMC10989754

